# Genetic Parameters and Genome-Wide Association Analysis of Reproductive and Calving Traits in Jersey Cattle

**DOI:** 10.3390/ani16193059

**Published:** 2026-09-29

**Authors:** Xubin Lu, Hao Zhu, Rui Cui, Huali Wu, Tianqi Zhao, Zhangping Yang

**Affiliations:** 1 College of Animal Science and Technology, Yangzhou University, Yangzhou 225009, China; lxb@yzu.edu.cn (X.L.); dx120240187@stu.yzu.edu.cn (H.Z.); 241902304@stu.yzu.edu.cn (R.C.); 241902321@stu.yzu.edu.cn (H.W.); 2 Zhejiang Key Laboratory of Cow Genetic Improvement and Milk Quality Research, Wenzhou 325000, China

**Keywords:** heritability, variance components, female fertility, candidate genes, KEGG pathway enrichment analysis

## Abstract

This study characterized the genetic basis of reproductive and calving traits in Jersey cattle at different physiological stages and identified potential candidate genes. After quality control, 2716 records were available for heifer reproductive traits, 2707 for multiparous cow reproductive traits, and 5423 calving records for calving ease (CE) and calf birth weight (CBW). CE and CBW were analyzed from the maternal perspective, with records assigned to the corresponding dams. Genetic parameters were estimated using AI-REML in DMU with animal, repeatability, and bivariate models. Genome-wide association analyses were performed using a mixed linear model based on 1,660,477 SNPs from 1578 genotyped Jersey cattle. Heritability estimates for age at first service (AFS) and age at first calving (AFC) were 0.416 and 0.380, respectively, whereas estimates for reproductive traits in multiparous cows were generally low (0.072–0.154). Calving interval (CI) and days open (DO) showed a strong positive genetic correlation (0.781). GWAS identified five significant loci associated with AFC, number of services per conception in heifers, CI, and dam-associated genetic variation in CE. Candidate genes included *PIK3C2G*, *DGKI*, *ABCC1*, *CCDC85A*, *CRMP1*, *EVC*, and *EVC2*. Exploratory KEGG enrichment suggested potential involvement of phosphatidylinositol signaling, Hedgehog signaling, ABC transporters, and inositol phosphate metabolism. Overall, reproductive traits showed distinct genetic characteristics between heifers and multiparous cows, and AFS and AFC exhibited relatively greater potential for genetic improvement. The identified genomic regions provide preliminary candidates for further validation and potential future application in genomic breeding.

## Simple Summary

Reproductive performance is important for the efficiency and sustainability of dairy cattle production. However, the genetic factors affecting reproduction may differ between young heifers and adult cows. In this study, we analyzed reproductive and calving records from Jersey cattle to investigate how genetics influences reproductive performance in heifers and multiparous cows. Age at first service and age at first calving showed a greater genetic contribution than most reproductive traits, suggesting that these traits may respond more effectively to genetic selection. By examining genetic differences across the genome, we also identified several regions associated with age at first calving, number of services in heifers, calving interval, and dam-associated genetic variation in calving ease. Several genes in these regions may be involved in reproductive processes, cell signaling, transport, and development. These findings improve our understanding of reproduction in Jersey cattle and may help support breeding strategies aimed at improving reproductive efficiency.

## 1. Introduction

Reproductive performance is a crucial functional trait affecting herd replacement efficiency, lactation continuity, productive lifespan, and the economic profitability of dairy farms. Conception failure, increased numbers of services per conception, and prolonged days open and calving intervals increase reproductive costs and may also increase involuntary culling, thereby reducing lifetime productivity [1,2]. Although sustained selection for milk yield has substantially improved dairy cattle productivity, unfavorable genetic relationships between production and fertility, together with negative energy balance, postpartum disorders, reduced estrous expression, impaired oocyte quality, and embryonic loss, can compromise reproductive performance in high-producing cows [2,3,4]. Reproductive traits generally have low heritability and are strongly affected by environmental and management factors, which limits the rate of genetic improvement; nevertheless, exploitable additive genetic variation allows continued selection for improved fertility and calving performance [5,6,7]. Therefore, reproductive efficiency has become an important component of modern dairy cattle breeding objectives alongside production, health, and longevity.

Jersey cattle are an important dairy breed, and their reproductive efficiency is directly linked to the supply of replacement heifers, herd expansion, and the sustainability of specialty dairy production systems. The breed originated on the Island of Jersey more than 200 years ago and was subsequently exported worldwide, resulting in the development of genetically differentiated populations under different production systems and breeding objectives [8]. The development of commercial Jersey herds in China has included the introduction of animals from established overseas populations; for example, a population of 449 Jersey cows imported from Australia in 2012 has been documented [9]. Consequently, Jersey herds maintained in China may differ in founder origin and subsequent selection history. The herd evaluated in the present study therefore represents a specific commercial population and should not be considered representative of all Jersey cattle maintained in China.

Previous studies in Australian Jersey herds have analyzed calving interval, calving-to-first-service interval, pregnancy status, and its predictor traits, revealing complex genetic associations between reproductive traits and lactation length, milk production performance, and survival traits [10]. Studies on Jersey crossbred cattle have shown that age at first calving, calving interval, days open, and number of services per conception generally exhibit low to moderate heritability, with varying degrees of genetic correlations among different reproductive traits and between reproductive and production traits [11]. Recent studies on Canadian Jersey cattle have further estimated genetic parameters for calving-related traits, including gestation length, calving ease, stillbirth, and calf size, and have combined genome-wide association and functional enrichment analyses to elucidate their underlying genetic basis [12]. Furthermore, whole-genome sequencing studies in Danish Jersey cattle and multi-breed GWAS indicate that QTL for reproductive traits in dairy cattle are, to some extent, breed- and population-specific [13,14,15]. Currently, genetic studies on reproductive and calving traits in Jersey cattle have been conducted predominantly in foreign populations. Differences in genetic background, selection history, allele frequencies, linkage disequilibrium structure, and reproductive management may limit the direct applicability of these findings to Chinese Jersey cattle. However, comprehensive studies jointly evaluating genetic parameters and genome-wide associations for reproductive and calving traits in Chinese Jersey populations remain limited.

The genetic expression of reproductive traits in dairy cattle may vary with physiological stage and parity. Previous studies have shown that reproductive traits expressed in heifers and multiparous cows may have different genetic parameters and correlation patterns [16,17,18]. Genetic correlations between corresponding or related reproductive traits measured in heifers and cows are generally lower than unity and may even be lower than those between parities in multiparous cows, indicating that reproductive performance in heifers and cows may not be genetically equivalent. Therefore, evaluating reproductive traits separately for heifers and multiparous cows may more accurately reflect the genetic variation and correlations within each reproductive group. Heritability and genetic correlations can reflect genetic variation at the population level and the shared genetic basis among traits, but cannot directly pinpoint specific genomic regions; GWAS can further identify genetic loci and candidate genes associated with reproductive and calving traits [19,20]. Given the typically complex polygenic genetic architecture of reproductive and calving traits in dairy cattle and the relatively limited effect of individual loci [21,22], integrating genetic parameter estimation with high-density genome-wide association analysis can therefore provide complementary quantitative and molecular insights into reproductive and calving traits evaluated in heifers and multiparous cows.

Based on the above research background, the objectives of this study were to estimate the heritability, genetic correlations, and phenotypic correlations of reproductive traits in heifers and multiparous cows, as well as calving-related traits; to identify genomic regions and potential candidate genes associated with these traits; and to explore potentially involved biological pathways, thereby providing population-specific genetic and genomic information for reproductive traits in Jersey cattle.

## 2. Materials and Methods

### 2.1. Study Population and Phenotypic Data

Data for this study were obtained from a large-scale Jersey cattle farm in Jiangsu Province. Service records, calving records, and pedigree information collected from 2018 to 2022 were used. Records from different sources were matched using individual animal IDs, and reproductive traits were derived from the recorded dates of birth, service, conception, and calving.

During the study period, the farm maintained an average inventory of approximately 3524 Jersey cattle, including approximately 2638 lactating cows. Animals were housed in free-stall barns, fed a total mixed ration three times daily, and milked three times daily. According to the herd production records, the average daily milk yield per lactating cow was 20.48 kg, with mean milk fat and milk protein contents of 4.98% and 4.19%, respectively. Periparturient cows were continuously monitored by on-duty personnel and a video-surveillance system, with direct inspections conducted at least every 1–2 h. Calving assistance was provided when no evident progress was observed within 60–90 min after the appearance of the fetal membranes or hooves, or when abnormal fetal presentation, prolonged labor, or obvious maternal exhaustion was observed. Calving-ease scores were recorded immediately after calving by the personnel attending the calving event and entered into the farm management system. Estrus was detected by visual observation two to three times daily in combination with continuous 24 h activity monitoring using pedometers or neck-mounted sensors. Animals showing spontaneous estrus were artificially inseminated approximately 8–12 h after the first observation of standing estrus or generation of an activity alert. Cows enrolled in the timed artificial insemination protocol received 100 μg of gonadotropin-releasing hormone (GnRH) on day 0, 25 mg of prostaglandin F_2_α (PGF_2_α) on day 7, and a second dose of 100 μg GnRH on day 9, followed by timed artificial insemination approximately 16 h after the second GnRH treatment. Pregnancy was initially diagnosed by transrectal B-mode ultrasonography at 30–33 d after insemination and reconfirmed at approximately 60 d after insemination. Cows were dried off with a target dry period of approximately 60 d before the expected calving date.

A total of 12 reproductive and calving traits were evaluated and grouped into three categories. (1) Heifer reproductive traits included age at first service (AFS), age at first calving (AFC), heifer gestation length (H-GL), and number of services per conception in heifers (H-AIS). (2) Multiparous cow reproductive traits included calving interval (CI), days open (DO), number of services per conception in multiparous cows (C-AIS), calving-to-first-service interval (C-ICF), gestation length in multiparous cows (C-GL), and age at calving in multiparous cows (C-AC). (3) Calving-related traits included calving ease (CE) and calf birth weight (CBW). The abbreviations, definitions, and units of all traits are presented in Table 1.

To ensure the reliability of the phenotypic data, quality control was applied to the raw records. Records with missing individual animal IDs or key reproductive dates, an abnormal chronological order of reproductive events, or insufficient information for calculating the corresponding traits were removed. The data were further screened using data-editing limits adapted from previously published studies for reproductive and calving traits in dairy cattle [23,24]: AFS, 270–900 d; AFC, 16–37 months; DO, 20–450 d; gestation length, 240–310 d; number of services, 1–9; interval between first and last service, 0–365 d; CBW, 15–50 kg; and CI, >280 d. These limits were used as data-editing criteria rather than clinical reference intervals.

After quality control, 2716 records were retained for the heifer reproductive traits, 2707 records for the multiparous cow reproductive traits, and 5423 calving records for CE and CBW. Pedigrees of animals with phenotypic records were traced using individual, sire, and dam IDs, and relationships among individuals were verified using genomic information. The final pedigree used for genetic parameter estimation comprised 10,745 animals, including phenotyped individuals and their traceable ancestors.

### 2.2. Genomic Data and Quality Control

Genotype data were available for 1631 Jersey cattle, all of which were included in the phenotypic population after quality control. Genomic DNA was extracted and genotyped with the GGP Bovine 100K SNP Chip by Neogen Bio-Scientific Technology (Shanghai) Co., Ltd (Shanghai, China)., using ARS-UCD1.2 (bosTau9) as the bovine reference genome. Haplotype phasing and genotype imputation were performed with Beagle (v5.2) using default parameters [25]. Genotypes from the GGP Bovine 100K SNP Chip were first imputed to BovineHD density using a reference panel comprising 3531 cattle provided by Aarhus University, Denmark. After imputation to the HD level, variants with a minor allele frequency (MAF) < 0.01 were excluded. The remaining variants were subsequently imputed to the whole-genome sequence level using a multi-breed reference panel comprising 3530 cattle from the CattleCell-GTEx database [26]. Genotype quality control was conducted using PLINK (v1.9) [27] according to the following criteria: (1) SNPs with a call rate > 90% were retained; (2) individuals with a genotype call rate > 95% were retained; (3) SNPs with minor allele frequency (MAF) < 0.05 were excluded; and (4) SNPs showing significant deviation from Hardy–Weinberg equilibrium (HWE) at *p* < 1.0 × 10^−6^ were excluded. Variants with Beagle dosage R^2^ (DR^2^) ≤ 0.80 were excluded after imputation. After quality control, 1578 Jersey cattle and 1,660,477 SNPs were retained for subsequent association analysis.

### 2.3. Genetic Parameter Estimation

Genetic parameters were estimated using the DMUAI module of the DMU software package (v5.6), employing the average information restricted maximum likelihood (AI-REML) algorithm [28]. Based on the data structure of each trait, a univariate animal model was used to estimate heritability for heifer reproductive traits, since each heifer had only a single valid record. For multiparous cow reproductive traits, as well as CE and CBW, which had repeated measurements across parities for the same cow, a univariate repeatability animal model was applied. Genetic and phenotypic correlations among heifer reproductive traits were estimated using bivariate animal models, whereas those among multiparous cow reproductive traits and calving-related traits were estimated using bivariate repeatability animal models. For CE and CBW, the animal effect represented the dam, and calf direct genetic effects and direct–maternal genetic covariance were not explicitly fitted. The subsequent GWAS used dam genotypes. Although the available calf pedigree information could in principle support direct–maternal analyses, these were not performed in the present study. The resulting estimates therefore describe dam-associated genetic variation under the fitted models rather than isolated maternal genetic effects. AFS, AFC, H-GL, and H-AIS were analyzed using a univariate animal model:

(1) $$\begin{array}{c} y=\mathrm{Xb}+\mathrm{Za}+e, \end{array}$$

where ***y*** is the vector of phenotypic observations; ***b*** is the vector of fixed effects; ***a*** is the vector of individual additive genetic effects; ***e*** is the vector of random residuals; and *X* and ***Z*** are the incidence matrices for fixed effects and additive genetic effects, respectively. For different heifer reproductive traits, appropriate systematic environmental factors were selected as fixed effects: for AFS and AFC, animal origin, birth month, and birth year were included; for H-GL, birth year and calving season were included; and for H-AIS, birth month and birth year were included. The additive genetic effects and random residuals were assumed to follow $a\sim N\left(0,A\sigma_{a}^{2}\right)$ and $e\sim N\left(0,I\sigma_{e}^{2}\right)$, where ***A*** is the additive genetic relationship matrix, ***I*** is an identity matrix, and $\sigma_{a}^{2}$ and $\sigma_{e}^{2}$ are the additive genetic variance and residual variance, respectively.

For CI, DO, C-AIS, C-ICF, C-GL, C-AC, CE, and CBW, repeated records across parities exist for the same cow; therefore, a repeatability animal model was applied:

(2) $$\begin{array}{c} y=\mathrm{Xb}+\mathrm{Za}+\mathrm{Wp}+e, \end{array}$$

where ***y*** is the vector of phenotypic observations; ***p*** is the vector of permanent environmental effects, ***W*** is the incidence matrix for permanent environmental effects, and the remaining terms are defined as in the univariate animal model. For each trait, appropriate factors were selected as fixed effects or covariates: for CI, birth year, calving year, parity, and calving season, were included as fixed effects; for DO, animal origin, birth year, calving year, parity, and calving season were included as fixed effects; for C-AIS, birth season, age at first calving, parity, calving year, and calving season were included as fixed effects; for C-ICF, calving year, calving season and age at first calving were included as fixed effects; for C-GL, animal origin, birth year, calving year, parity, calving season, and age at first calving were included as fixed effects; for C-AC, birth year, birth season, animal origin, parity, calving season, and age at first calving were included as fixed effects; for CE, parity, calving year, calving season, calf sex, and calf birth status were included as fixed effects, with CBW fitted as a covariate; for CBW, animal origin, parity, calf sex, calving year, calving season, age at first calving, and calf birth status were included as fixed effects. The additive genetic effects, permanent environmental effects, and random residuals in the repeatability model were assumed to follow $a\sim N\left(0,A\sigma_{a}^{2}\right)$, $p\sim N\left(0,I\sigma_{p}^{2}\right)$, and $e\sim N\left(0,I\sigma_{e}^{2}\right)$, respectively, where ***A*** is the additive genetic relationship matrix, ***I*** is an identity matrix, and $\sigma_{a}^{2}$, $\sigma_{p}^{2}$ and $\sigma_{e}^{2}$ are the additive genetic variance, permanent environmental, and residual variance, respectively.

Genetic and phenotypic correlations among heifer reproductive traits, multiparous cow reproductive traits, and calving-related traits were all estimated using bivariate models. For any two traits *i* and *j*, the general bivariate animal model is expressed as:

(3) $$\begin{array}{c} \left[\begin{array}{c} y_{i}\\ y_{j} \end{array}\right]=\left[\begin{array}{cc} X_{i} & 0\\ 0 & X_{j} \end{array}\right]\left[\begin{array}{c} b_{i}\\ b_{j} \end{array}\right]+\left[\begin{array}{cc} Z_{i} & 0\\ 0 & Z_{j} \end{array}\right]\left[\begin{array}{c} a_{i}\\ a_{j} \end{array}\right]+\left[\begin{array}{c} e_{i}\\ e_{j} \end{array}\right], \end{array}$$

where *y_i_* and *y_j_* are the vectors of phenotypic observations for traits *i* and *j*, respectively; *b_i_* and *b_j_* are the vectors of fixed effects for the two traits; *a_i_* and *a_j_* are the vectors of additive genetic effects for the two traits; *e_i_* and *e_j_* are the vectors of random residuals for the two traits; and *X_i_*, *X_j_*, *Z_i_*, and *Z_j_* are the corresponding incidence matrices. The fixed effects fitted for each trait in the bivariate analyses were generally consistent with those used in the corresponding univariate models. However, for the bivariate analysis between CE and CBW, CBW was not fitted as a covariate in the CE equation, thereby allowing the genetic and phenotypic covariances between the two traits to be estimated without conditioning CE on CBW.

Variance structure of the additive genetic effects for the two traits:

(4) $$\begin{array}{c} \mathrm{Var}\left(\begin{array}{c} a_{i}\\ a_{j} \end{array}\right)=\left(\begin{array}{cc} \sigma_{a_{i}}^{2} & \sigma_{a_{\mathrm{ij}}}\\ \sigma_{a_{\mathrm{ij}}} & \sigma_{a_{j}}^{2} \end{array}\right)⊗A \end{array}$$

Variance structure of the random residuals:

(5) $$\begin{array}{c} \mathrm{Var}\left(\begin{array}{c} e_{i}\\ e_{j} \end{array}\right)=\left(\begin{array}{cc} \sigma_{e_{i}}^{2} & \sigma_{e_{\mathrm{ij}}}\\ \sigma_{e_{\mathrm{ij}}} & \sigma_{e_{j}}^{2} \end{array}\right)⊗I, \end{array}$$

where $\sigma_{a_{i}}^{2}$ and $\sigma_{a_{j}}^{2}$ are the additive genetic variances of traits *i* and *j*, respectively, and $\sigma_{a_{\mathrm{ij}}}$ is the additive genetic covariance between the two traits; $\sigma_{e_{i}}^{2}$ and $\sigma_{e_{j}}^{2}$ are the residual variances of the two traits, and $\sigma_{e_{\mathrm{ij}}}$ is the residual covariance between the two traits, ***A*** is the additive genetic relationship matrix, and ***I*** is an identity matrix.

For multiparous cow reproductive traits with repeated records, as well as CE and CBW, individual permanent environmental effects were further incorporated into the aforementioned bivariate animal model to construct a bivariate repeatability animal model:

(6) $$\begin{array}{c} \left[\begin{array}{c} y_{i}\\ y_{j} \end{array}\right]=\left[\begin{array}{cc} X_{i} & 0\\ 0 & X_{j} \end{array}\right]\left[\begin{array}{c} b_{i}\\ b_{j} \end{array}\right]+\left[\begin{array}{cc} Z_{i} & 0\\ 0 & Z_{j} \end{array}\right]\left[\begin{array}{c} a_{i}\\ a_{j} \end{array}\right]+\left[\begin{array}{cc} W_{i} & 0\\ 0 & W_{j} \end{array}\right]\left[\begin{array}{c} p_{i}\\ p_{j} \end{array}\right]+\left[\begin{array}{c} e_{i}\\ e_{j} \end{array}\right], \end{array}$$

where *p_i_* and *p_j_* are the vectors of permanent environmental effects for traits i and j, respectively, and *W_i_* and *W_j_* are the corresponding incidence matrices. The variance structures of the additive genetic effects and random residuals are the same as those in the bivariate animal model described above. The variance structure of the permanent environmental effects is:

(7) $$\begin{array}{c} \mathrm{Var}\left(\begin{array}{c} p_{i}\\ p_{j} \end{array}\right)=\left(\begin{array}{cc} \sigma_{{\mathrm{pe}}_{i}}^{2} & \sigma_{{\mathrm{pe}}_{\mathrm{ij}}}\\ \sigma_{{\mathrm{pe}}_{\mathrm{ij}}} & \sigma_{{\mathrm{pe}}_{j}}^{2} \end{array}\right)⊗I \end{array}$$

Based on the estimated variance and covariance components from the models, heritability, genetic correlations, and phenotypic correlations were calculated for each reproductive trait. For heifer reproductive traits analyzed using the univariate animal model, the heritability of trait was calculated as:

(8) $$\begin{array}{c} h_{i}^{2}=\frac{\sigma_{a_{i}}^{2}}{\sigma_{a_{i}}^{2}{+\sigma }_{e_{i}}^{2}} \end{array}$$

For multiparous cow reproductive traits, CE, and CBW analyzed using the repeatability animal model, the heritability of trait was calculated as:

(9) $$\begin{array}{c} h_{i}^{2}=\frac{\sigma_{a_{i}}^{2}}{\sigma_{a_{i}}^{2}{+\sigma_{{\mathrm{pe}}_{i}}^{2}+\sigma }_{e_{i}}^{2}}, \end{array}$$

where $h_{i}^{2}$ is the heritability of trait *i*; $\sigma_{a_{i}}^{2}$, $\sigma_{{\mathrm{pe}}_{i}}^{2}$, and $\sigma_{e_{i}}^{2}$ are the additive genetic variance, permanent environmental variance of trait *i*, and residual variance of trait *i*, respectively. For CE and CBW, the additive genetic effect corresponds to the dam. The genetic correlation between traits *i* and *j* was calculated as:

(10) $$\begin{array}{c} r_{g\left(\mathrm{ij}\right)}=\frac{\sigma_{a\left(\mathrm{ij}\right)}}{\sqrt{\sigma_{a\left(i\right)}^{2}\sigma_{a\left(j\right)}^{2}}}, \end{array}$$

where *r_g_*_(*ij*)_ is the genetic correlation between traits *i* and *j*; *σ_a_*_(*ij*)_ is the additive genetic covariance between the two traits; and $\sigma_{a\left(i\right)}^{2}$ and $\sigma_{a\left(j\right)}^{2}$ are the additive genetic variances of traits *i* and *j*, respectively. The phenotypic correlation between any two reproductive traits *i* and *j* was calculated as:

(11) $$\begin{array}{c} r_{p\left(\mathrm{ij}\right)}=\frac{\sigma_{p\left(\mathrm{ij}\right)}}{\sqrt{\sigma_{p\left(i\right)}^{2}\sigma_{p\left(j\right)}^{2}}}, \end{array}$$

where *r_p_*_(*ij*)_ is the phenotypic correlation between traits i and j; *σ_p_*_(*ij*)_ is the phenotypic covariance between the two traits; and $\sigma_{p\left(i\right)}^{2}$ and $\sigma_{p\left(j\right)}^{2}$ are the phenotypic variances of traits *i* and *j*, respectively.

The statistical significance of genetic correlations was assessed using approximate two-sided Wald tests, with Z calculated as the estimated genetic correlation divided by its standard error. Correlations were considered significantly different from zero at *p* < 0.05.

### 2.4. Genome-Wide Association Analysis

Estimated breeding values (EBVs) and their corresponding prediction error variances were obtained from the genetic evaluation described in Section 2.3. Following Garrick et al. [29], parental-average information was removed from the EBVs before deregression. The reliability of the EBV for animal *i* was calculated as:

(12) $$\begin{array}{c} r_{i}^{2}=1-\frac{{\mathrm{PEV}}_{i}}{\sigma_{a}^{2}} \end{array}$$

where *PEV_i_* is the prediction error variance and $\sigma_{a}^{2}$ is the additive genetic variance of the corresponding trait.

The deregressed proof was subsequently calculated as:

(13) $$\begin{array}{c} {\mathrm{DRP}}_{i}=\frac{{{\mathrm{EBV}}^{*}}_{i}}{r_{i}^{2}} \end{array}$$

where ${{\mathrm{EBV}}^{*}}_{i}$ denotes the EBV after removal of parental-average information, and $r_{i}^{2}$ denotes its corresponding reliability after this adjustment. The resulting DRP values served as response variables in the subsequent genome-wide association analysis. No additional reliability-based weighting was applied to these values in the GWAS model, which assumed homogeneous residual variance across individuals.

Genome-wide association analysis was performed using the DRP values described above as response variables. Genome-wide association analysis was performed using the mixed linear model association (MLMA) implemented in GCTA (v 1.94.0) [30], with the DRP values described above used as response variables. Each SNP was fitted as a fixed effect, while the genomic relationship matrix was incorporated to account for genomic relatedness among individuals and potential population structure. The model was expressed as:

(14) $$\begin{array}{c} y_{\mathrm{DRP}}=1\mu +\mathrm{m\beta}+g+e, \end{array}$$

where ***y_DRP_*** is the vector of deregressed proofs; ***μ*** is the overall mean; **1** is a vector of ones; ***m*** is the vector of genotypes for the SNP being tested; ***β*** is the allele substitution effect of the SNP being tested; ***g*** is the vector of random polygenic effects; and ***e*** is the vector of random residuals. The random polygenic effects and random residuals were assumed to follow $g\sim N\left(0,K\sigma_{g}^{2}\right)$ and $e\sim N\left(0,I\sigma_{e}^{2}\right)$, respectively, where *K* is the genomic relationship matrix, *I* is an identity matrix, and $\sigma_{g}^{2}$ and $\sigma_{e}^{2}$ are the variances of the random polygenic effects and residuals, respectively.

Association tests were performed for each SNP across the genome. The Bonferroni correction was applied to control the genome-wide false positive rate, and the genome-wide significance threshold was set at 3.01 × 10^−8^ (0.05/1,660,477), and 6.02 × 10^−9^ (0.01/1,660,477).

### 2.5. Candidate Gene Annotation and Enrichment Analysis

Candidate gene annotation was performed for SNPs reaching genome-wide significance. Based on the ARS-UCD1.2 bovine reference genome, genes located within ±200 kb of each significant SNP were considered positional candidate genes, whereas genes harboring significant SNPs were directly included as candidate genes. The resulting candidate gene set was subjected to exploratory Kyoto Encyclopedia of Genes and Genomes (KEGG) pathway enrichment analysis using the Database for Annotation, Visualization and Integrated Discovery (DAVID) [31] to identify potentially enriched biological pathways.

## 3. Results

### 3.1. Descriptive Statistics

The descriptive statistics of the reproductive and calving traits are presented in Table 2. Heifers had mean AFS and AFC values of 480.98 d and 25.43 months, respectively. In multiparous cows, CI and DO averaged 377.41 and 97.88 d, respectively. Calving difficulty was generally low, with a mean CE score of 2.08, while mean CBW was 28.88 kg. Greater phenotypic variation was observed for AFS, CI, DO, and C-AC, whereas C-GL showed relatively limited variation.

### 3.2. Genetic Parameters of Reproductive and Calving Traits

The heritability estimates for heifer reproductive traits ranged from 0.133 to 0.416 (Table 3). AFS (0.416 ± 0.045) and AFC (0.380 ± 0.052) showed higher heritability than H-GL and H-AIS. Moderate to high genetic correlations were observed among several heifer reproductive traits, particularly between AFC and H-AIS (0.862), AFS and H-AIS (0.780), and AFS and AFC (0.602). Phenotypically, AFS and AFC were positively correlated (0.780, *p* < 0.05).

Heritability estimates for reproductive traits in multiparous cows were generally low, ranging from 0.072 to 0.154 (Table 4). C-GL and C-ICF showed the highest estimates, whereas C-AIS had the lowest. CI and DO exhibited a very strong genetic correlation (0.781), and both traits were also strongly genetically correlated with C-AIS (0.737 and 0.756, respectively). Similar relationships were observed at the phenotypic level.

Under the dam-based models, the heritability estimate for CBW was 0.190 ± 0.043, which was higher than the corresponding estimate for CE (0.028 ± 0.015) (Table 5). The genetic correlation between CE and CBW was −0.127, and their phenotypic correlation was 0.123 (*p* < 0.05).

### 3.3. Genome-Wide Association Signals for Reproductive and Calving Traits

**Figure 1:**
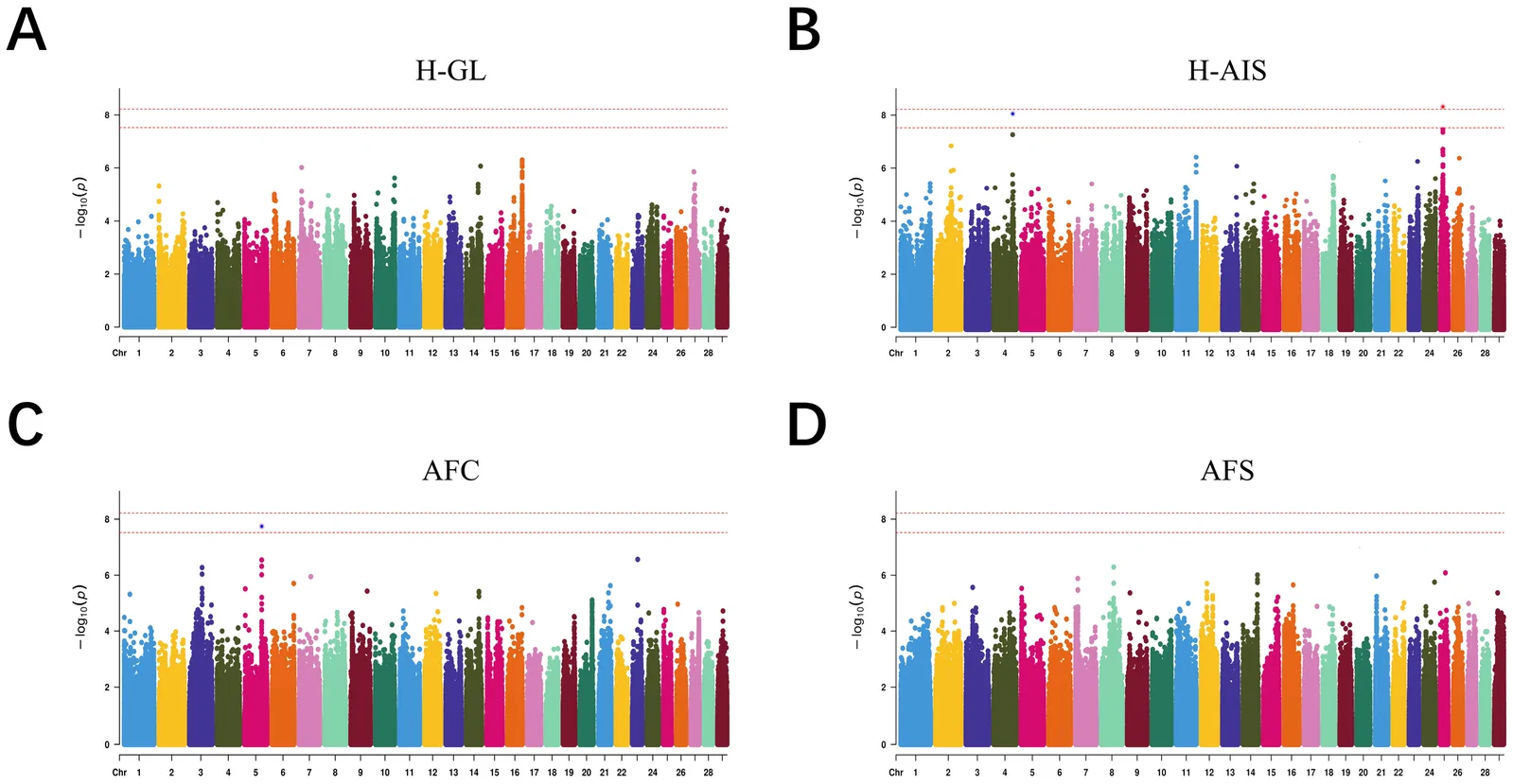
Manhattan plots of GWAS for reproductive traits in heifers. (**A**–**D**) represent the GWAS results for H-GL, H-AIS, AFC, and AFS, respectively. The *x*-axis represents bovine autosomes, and the *y*-axis represents −log_10_ (*p*-value) for each SNP. The two red dotted lines above and below represent the genome-wide significance threshold after Bonferroni correction, and the corresponding *p*-values are 6.02 × 10^−9^ (0.01/1,660,477), and 3.01 × 10^−8^ (0.05/1,660,477).

**Figure 2:**
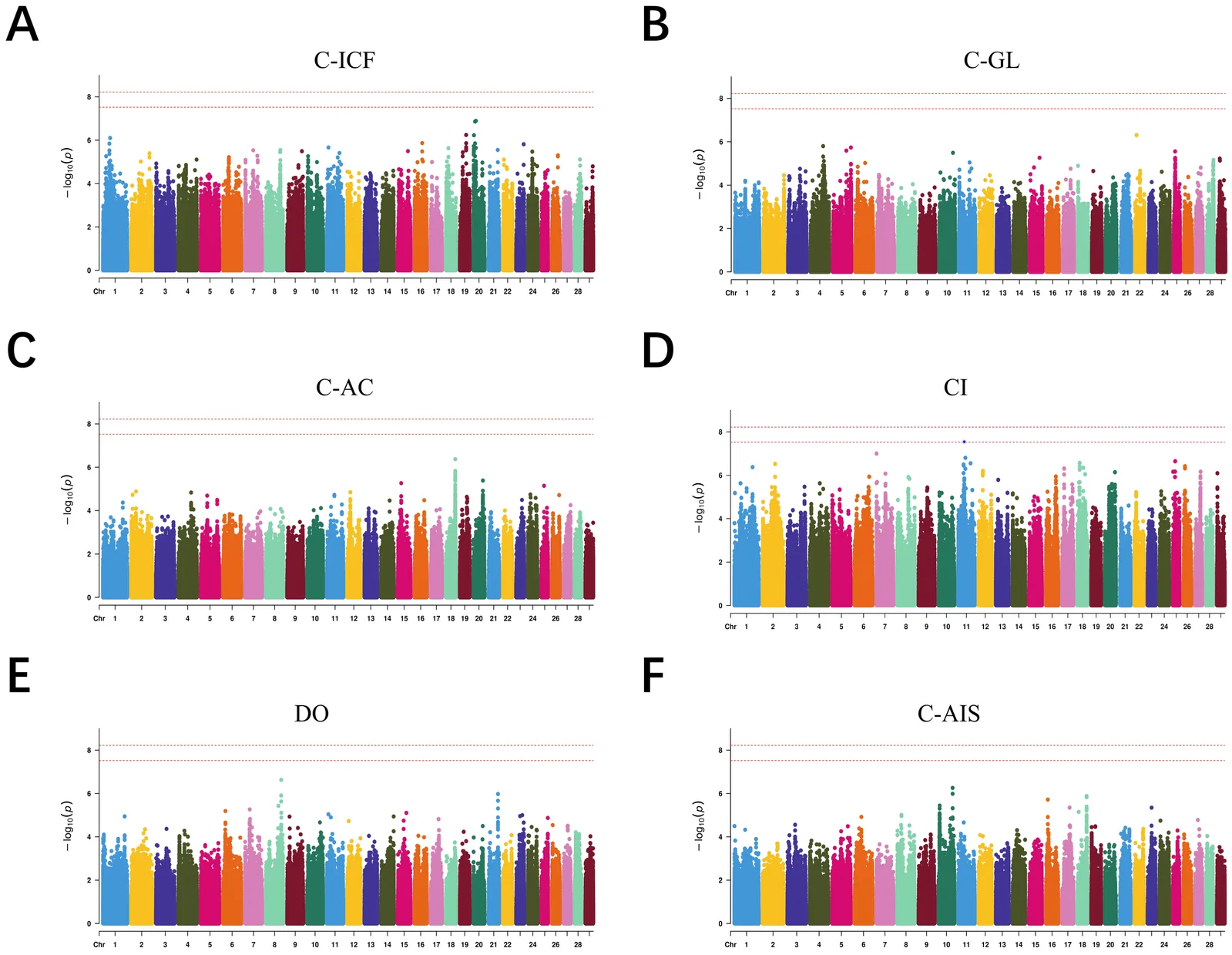
Manhattan plots of GWAS for reproductive traits of multiparous cows. (**A**–**F**) represent the GWAS results for C-ICF, C-GL, C-AC, CI, DO, and C-AIS, respectively. The *x*-axis represents bovine autosomes, and the *y*-axis represents −log_10_(*p*-value) for each SNP. The two red dotted lines above and below represent the genome-wide significance threshold after Bonferroni correction, and the corresponding *p*-values are 6.02 × 10^−9^ (0.01/1,660,477), and 3.01 × 10^−8^ (0.05/1,660,477).

**Figure 3:**
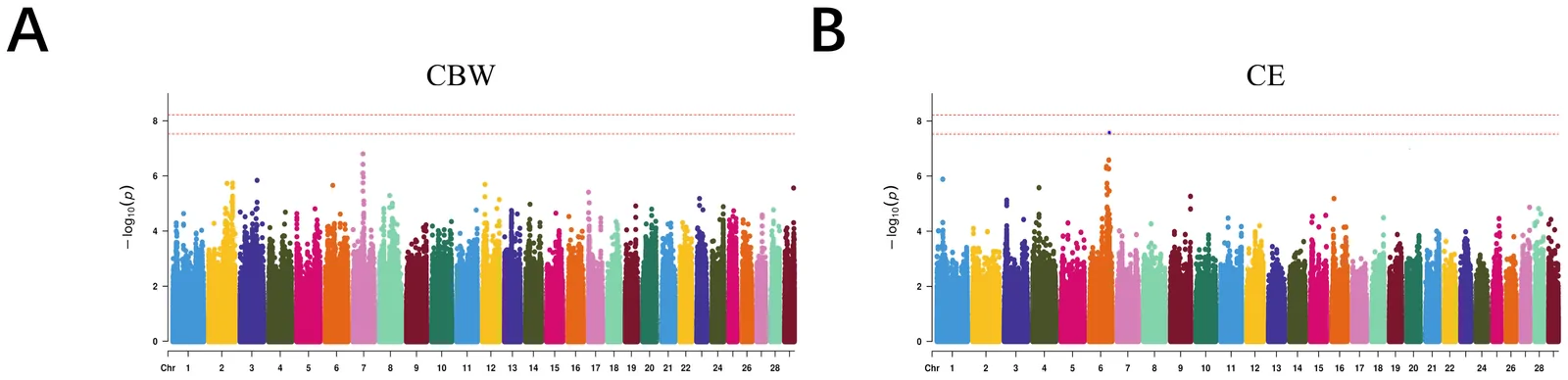
Manhattan plots of GWAS for calving traits in Jersey cattle. (**A**) and (**B**) represent the GWAS results for CBW and CE, respectively. The *x*-axis represents bovine autosomes, and the *y*-axis represents −log_10_(*p*-value) for each SNP. The CE and CBW analyses used dam genotypes and represent associations detected using dam genotypes. The two red dotted lines above and below represent the genome-wide significance threshold after Bonferroni correction, and the corresponding *p*-values are 6.02 × 10^−9^ (0.01/1,660,477), and 3.01 × 10^−8^ (0.05/1,660,477).

The Q–Q plots showed that the observed *p*-value distributions generally followed the expected distributions under the null hypothesis for most traits, with deviations mainly occurring in the upper tails (Figure S1). Genome-wide significant associations were identified for AFC, H-AIS, CI, and dam-associated genetic variation in CE, with five lead loci located on chromosomes 4, 5, 6, 11, and 25 (Figure 1, Figure 2 and Figure 3 and Table 6). No genome-wide significant associations were detected for the remaining reproductive and calving traits.

For reproductive traits in heifers, the significant association signal for AFC was located near 91,433,413 bp on chromosome 5, where candidate genes including *CAPZA3*, *PLCZ1*, and *PIK3C2G* were annotated (Figure 1C and Table 6). Significant association signals for H-AIS were detected on chromosomes 4 and 25. The region near 101,232,152 bp on chromosome 4 contained *DGKI*, whereas the region near 14,372,905 bp on chromosome 25 contained *MYH11*, *CEP20*, *ABCC1*, and *ABCC6* (Figure 1B and Table 6).

For reproductive traits in cows, only CI showed a genome-wide significant association signal, which was located near 39,069,189 bp on chromosome 11, with *CCDC85A* annotated in this region (Figure 2D and Table 6). Among the calving-related traits, the GWAS using dam genotypes identified an association with CE near 103,385,828 bp on chromosome 6, where *CRMP1*, *EVC*, and *EVC2* were annotated as positional candidates. No genome-wide significant association with CBW was detected using dam genotypes (Figure 3 and Table 6).

### 3.4. KEGG Pathway Enrichment Analysis of Candidate Genes

**Figure 4:**
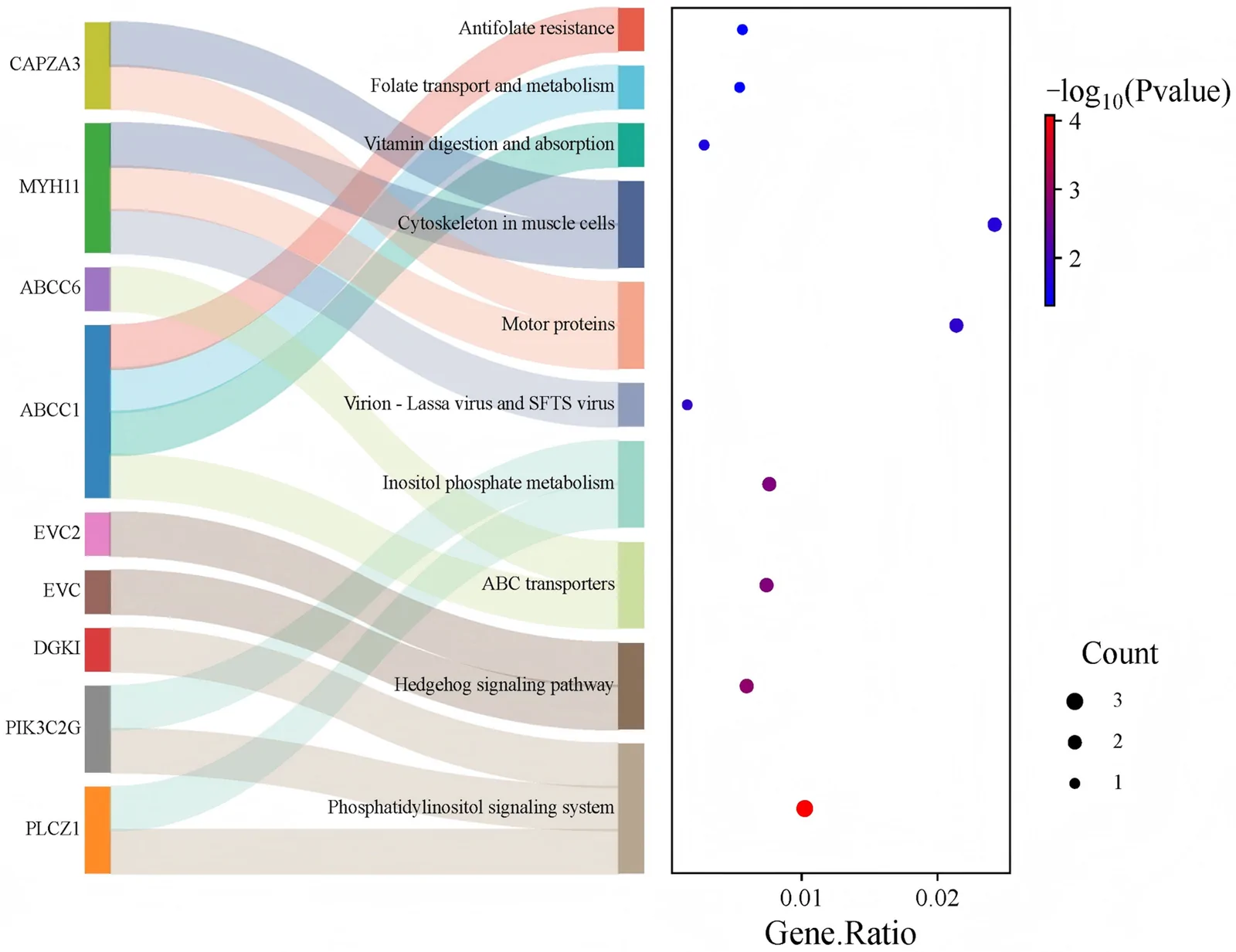
KEGG pathway enrichment analysis of candidate genes identified by GWAS for reproductive and calving-related traits in Jersey cattle. The Sankey diagram on the left shows the relationships between candidate genes and enriched KEGG pathways, with links indicating the assignment of genes to the corresponding pathways. The bubble plot on the right shows the Gene Ratio and enrichment significance of each pathway. Bubble size represents the number of candidate genes enriched in each pathway (Count), and color represents −log_10_ (*p*-value), with higher values indicating greater enrichment significance.

Exploratory KEGG enrichment analysis of the 12 candidate genes identified 10 enriched pathways (Figure 4). The phosphatidylinositol signaling system, inositol phosphate metabolism, Hedgehog signaling pathway, ABC transporters, and cytoskeleton-related pathways were among the categories potentially represented in this candidate gene set and warrant further validation.

## 4. Discussion

The reproductive and calving phenotypes observed in the present Jersey population were generally comparable with those reported for other Jersey populations, although differences among populations were evident. The mean AFC was 25.43 months, whereas the mean CI and DO in multiparous cows were 377.41 and 97.88 d, respectively. The mean AFC in the present study falls within the 25–26-month category reported for pedigree Jersey heifers in the United Kingdom; heifers in that category had a subsequent mean CI of 391 d [32]. In comparison, AFC, CI, and DO average 1060.06 d, 440.45 d, and 167.86 d, respectively, in an Indian Jersey crossbred population [11]. However, the Indian population was crossbred and maintained under different climatic and production conditions and therefore is not directly comparable with the present population. Differences in these traits among populations may reflect breed composition, season, nutritional status, herd management, reproductive management, and data-editing procedures [33,34]. In particular, postpartum metabolic and physiological conditions may contribute to variation in CI and DO because negative energy balance can delay ovarian cyclicity and subsequent conception in lactating cows [35]. Calving difficulty was relatively low in the present population, with a mean CE score of 2.08. Nevertheless, direct numerical comparison with other Jersey populations is limited because calving difficulty may be reported using different scoring definitions and category distributions. Previous comparisons among Holstein, Jersey, and crossbred cattle have also demonstrated that calving performance is influenced by breed background as well as maternal, fetal, and environmental factors [36]. Therefore, the reproductive and calving performance observed here should be interpreted within the genetic, management, and environmental context of the herd evaluated in this study.

AFS and AFC had relatively high heritability estimates of 0.416 and 0.380, respectively, whereas H-GL, H-AIS, and most reproductive traits in multiparous cows had low heritability estimates. The estimates for AFS and AFC were higher than those generally reported for female fertility traits in dairy cattle [37,38] and were also higher than the AFC heritability of 0.12–0.15 reported for Indian Jersey crossbred cattle [11]. By contrast, the low heritability estimates obtained for the multiparous cow reproductive traits were more consistent with the estimates reported in that population for CI (0.05–0.08), DO (0.08–0.09), and number of services per conception (0.04–0.06) [11]. Heritability estimates for calving-related traits in Canadian Jersey cattle were also generally low, ranging from 0.01 to 0.11 [12]. Accordingly, the low heritability estimated for CE under the dam-based model (0.028) is consistent with the general pattern reported for Jersey calving traits. The heritability estimated for CBW under the dam-based model (0.190) was comparatively higher; however, direct comparison is limited because the Canadian study evaluated ordinal calf size rather than measured birth weight, separated heifers and cows, and applied different definitions of direct and maternal genetic effects [12]. Several factors may have contributed to the relatively high heritability estimates obtained for AFS and AFC. First, the data were derived from a single commercial farm with relatively uniform feeding and reproductive management, which may have reduced environmental heterogeneity and affected the partitioning of phenotypic variance. Second, the heifer-trait dataset comprised 2716 records. Although this sample size does not necessarily result in upwardly biased estimates, it may increase the sensitivity of variance-component estimates to sampling variation and the representation of individual families. The standard errors of 0.045 for AFS and 0.052 for AFC should therefore be considered when interpreting the corresponding point estimates. Third, the pedigree used for genetic evaluation comprised 10,745 animals, and genomic information was used to verify the recorded relationships; however, the variance components were estimated using a pedigree-based relationship matrix. Unequal family representation, close relationships among animals, and the founder structure of this single herd may therefore have influenced the partitioning of additive genetic and residual variance. Fourth, the exclusion of incomplete or chronologically inconsistent records and the application of predefined data-editing limits improved data reliability but may also have restricted the phenotypic distribution and affected the estimated genetic and residual variances. Finally, the models for AFS and AFC accounted for animal source, birth month, and birth year, whereas other potentially relevant individual-level factors, including heifer growth, body weight, health status, estrus-detection accuracy, and insemination decisions, were unavailable or not explicitly modeled. Differences in population structure, data-editing procedures, and model specification may therefore partly explain the differences between the present estimates and those reported previously. Consequently, the AFS and AFC estimates should be interpreted as specific to the population, dataset, and analytical model used in this study rather than generalized directly to other Jersey populations.

Among heifer traits, AFC showed a high positive genetic correlation with H-AIS (0.862), followed by AFS with H-AIS (0.780), whereas AFS and AFC showed a moderate-to-high positive genetic correlation (0.602). This general pattern is consistent with the positive genetic correlations among reproductive traits reported in Jersey crossbred cattle, which ranged from 0.05 to 0.98, except for the correlation between AFC and number of services per conception [11]. Because AFS, AFC, and H-AIS are linked through service, conception, and calving dates, some of these correlations may reflect temporal and mathematical dependencies rather than solely shared genetic mechanisms. In multiparous cows, CI and DO exhibit a strong positive genetic correlation (0.781), consistent with their closely related biological and mathematical definitions because DO represents a major component of the interval between consecutive calvings. This strong correlation should therefore be interpreted partly in the context of the mathematical relationship between the two traits rather than solely as evidence of shared biological pathways. In Australian Jersey herds, CI was also reported to have high genetic correlations (>0.7) with calving-to-first-service interval, pregnancy status, and lactation length [10], indicating that CI may be genetically associated with several temporally related reproductive measures. C-AC is partly mathematically dependent on age at first calving, parity, and previous reproductive intervals; therefore, its genetic parameters should be interpreted cautiously.

For the calving traits, CE and CBW showed a weak negative genetic correlation (−0.127) under the dam-based model. An important limitation of the calving-trait analyses is that calf direct and maternal genetic effects were not explicitly separated. For CE and CBW, the fitted models assigned calving records to dams and therefore estimated dam-associated genetic variation. Although pedigree information was available for the calves and could in principle support pedigree-based direct–maternal analyses, calf direct effects were not fitted in the present study, and calf genotypes were unavailable for genomic analysis. Calf direct genetic effects may influence both CE and CBW through fetal growth, body size, and morphology, and failure to partition direct and maternal genetic components and their covariance may affect estimates of genetic variance, heritability, and breeding values [39]. Consequently, the genetic parameter estimates for CE and CBW and the significant GWAS association identified for CE should be interpreted within the fitted dam-based framework rather than as evidence of isolated maternal genetic effects.

Although CE was recorded on a four-category ordinal scale, it was analyzed using a linear repeatability model to maintain a consistent analytical framework across the calving traits and to facilitate the estimation of dam-associated additive genetic and permanent environmental effects. Vanderick et al. [40] reported direct heritability estimates of approximately 0.08 and 0.12 using linear and threshold models, respectively, whereas the corresponding maternal heritability estimates were approximately 0.02 and 0.04. Although the two approaches showed generally small differences in overall predictive performance, differences in breeding-value rankings were more evident for sires with fewer progeny records [40]. The heritability estimated under the dam-based model (0.028) was numerically close to the estimates reported in that study, although direct comparison is limited by differences in population, model structure, and estimation scale. Nevertheless, a linear model assumes equal distances between adjacent CE categories and normally distributed residuals, whereas a threshold model assumes an underlying continuous liability. Consequently, the variance components and heritability estimated here are expressed on the observed scale and may be sensitive to the frequencies of the different CE categories. The use of a threshold model could therefore produce different genetic parameter estimates, rankings of dam breeding values, and DRP values, potentially affecting the magnitude or significance of the GWAS associations. The CE estimates and associated loci identified in this study should thus be interpreted with caution and validated using threshold-model analyses in larger independent populations.

The GWAS results further suggested a trait-specific genetic architecture for reproductive performance, consistent with previous genome-wide association studies showing substantial heterogeneity in associated regions across fertility and reproductive traits [41,42,43]. For AFC, the significant region on chromosome 5 contained *CAPZA3*, *PLCZ1*, and *PIK3C2G*. *PLCZ1* plays an important role in sperm-mediated oocyte activation, whereas *CAPZA3* has been associated with spermatogenesis and sperm function [44,45]. Because these functional observations are primarily derived from male reproductive biology, they should not be interpreted as direct evidence that *CAPZA3* or *PLCZ1* regulates AFC in females. Instead, they indicate that the associated region contains genes with broader reproductive relevance. *PIK3C2G* may provide a more biologically plausible link to female reproductive processes because it belongs to the phosphoinositide 3-kinase family, and PI3K signaling has been implicated in bovine oocyte meiotic maturation [46]. This observation is also consistent with the enrichment of the phosphatidylinositol signaling system and inositol phosphate metabolism pathways in the present study. For H-AIS, significant regions contained *DGKI*, *MYH11*, *CEP20*, *ABCC1*, and *ABCC6*. *DGKI* is involved in the conversion of diacylglycerol to phosphatidic acid and therefore participates in lipid-mediated cellular signaling [47]; however, direct evidence linking *DGKI* to female fertility in cattle remains limited. *ABCC1* belongs to the ABC transporter family and has been associated with transport processes in reproductive tissues. Although this evidence was obtained in pigs [48], it suggests a potential role for ABC-mediated transport during reproductive processes. Studies in mice have also implicated *MYH11*-related smooth-muscle processes in uterine function [49]. Taken together, these findings suggest that H-AIS-associated regions may involve several biological processes, including cellular signaling, membrane transport, and smooth-muscle function, although direct functional validation in cattle is still required.

Among reproductive traits in multiparous cows, the significant region associated with CI on chromosome 11 contained *CCDC85A*. At present, direct evidence linking *CCDC85A* to female fertility or calving interval in cattle remains limited. Therefore, this gene should be considered a preliminary positional candidate, and its potential role in reproductive regulation requires further validation in independent populations and functional studies. For CE, the dam-genotype GWAS identified a genomic region associated with CE on chromosome 6 containing the positional candidates *CRMP1*, *EVC*, and *EVC2*. *EVC* and *EVC2* encode components involved in primary cilium-mediated Hedgehog signal transduction [50,51], and the exploratory enrichment suggests that Hedgehog signaling may be involved in maternal calving performance. However, this association does not establish a causal role for these genes or imply a direct calf genetic effect. The candidate genes identified here differed from those reported for calving-related traits in Canadian Jersey cattle, which included *MTHFR*, *SERPINA5*, *IGFBP3*, and *ZRANB1* [12]. This limited overlap may reflect differences in trait definitions, direct and maternal genetic models, marker density, linkage disequilibrium patterns, and population structure and further supports the need for independent validation of the present associations.

Genome-wide significant association signals were detected only for AFC, H-AIS, CI, and CE, whereas no significant loci were identified for the remaining reproductive and calving traits at the current significance threshold. This absence of significant associations does not imply that these traits lack a genetic basis, but may instead reflect the complex genetic architecture of reproductive traits and the limited statistical power of GWAS. Previous studies have shown that, even within the same population and analytical framework, the number of significant loci detected can vary considerably among reproductive traits, and some traits may fail to yield associations reaching stringent genome-wide significance thresholds [52]. In addition, the overlap of reproduction-related QTL across populations is often limited, suggesting substantial population- and trait-specificity in the genetic architecture of reproductive performance [53]. Longitudinal GWAS have further indicated that the genetic effects underlying reproductive traits may vary across parities and physiological stages, suggesting a degree of stage-dependent genetic regulation [54]. Therefore, for reproductive traits characterized by low heritability and a polygenic architecture involving numerous small-effect variants, limited sample size and stringent genome-wide multiple-testing correction may further reduce the power to detect true associations with small effects. The GWAS results may likewise be influenced by the genetic structure, allele frequencies, and linkage disequilibrium patterns of this herd. Although relatedness among animals was accounted for in the mixed linear model, this does not fully overcome the limited population representativeness. Validation in larger, independent Jersey populations from multiple farms and regions is therefore required before these estimates and associated loci can be applied in broader breeding programs.

Several limitations of this study should be acknowledged. The phenotypic data came from a single-farm Jersey population, and the relatively limited GWAS sample size may have reduced power, particularly for low-heritability traits. CE and CBW were analyzed using dam-associated models without separating calf direct and maternal genetic effects or their covariance. Because the objective of this study was to characterize dam-associated genetic variation and perform GWAS based on dam genotypes, direct–maternal decomposition was not included in the present analytical framework. Therefore, the estimated parameters and genomic associations should be interpreted as reflecting dam-associated genetic variation under the fitted models, and unpartitioned calf direct effects may have influenced the results. Candidate genes were based on ±200 kb positional annotation, which does not establish causality, and the exploratory KEGG analysis relied on a relatively small candidate-gene set. The associations also lacked independent population and functional validation. Nevertheless, this study provides population-specific genetic estimates and preliminary genomic evidence for reproductive and calving traits in the Jersey population evaluated here, warranting validation in larger independent populations.

## 5. Conclusions

This study revealed distinct genetic characteristics of reproductive traits between Jersey heifers and multiparous cows. Age at first service and age at first calving exhibited relatively high heritability, indicating greater potential for genetic improvement than the reproductive traits evaluated in multiparous cows. The genomic regions associated with age at first calving, number of services per conception in heifers, calving interval, and calving ease under the dam-based model provide preliminary targets for further investigation. Collectively, these findings may inform stage-specific trait prioritization in Jersey breeding programs, although validation in larger, independent, and multi-herd populations is required before practical application.

## Figures and Tables

**Table 1:** Names, abbreviations, and definitions of reproductive and calving traits evaluated in the study.

Item	Abbreviation	Definition
Age at first service, d	AFS	Interval from birth date to date of first service
Age at first calving, months	AFC	Interval from birth date to date of first calving
Heifer gestation length, d	H-GL	Interval from the conception date to the date of first calving in heifers
Heifer number of services, times	H-AIS	Number of services from first service to conception in heifers
Calving interval, d	CI	Interval between two consecutive calving dates
Days open, d	DO	Interval from calving date to subsequent conception date in multiparous cows
Multiparous cow number of services, times	C-AIS	Number of services from first service to conception within a reproductive cycle in multiparous cows
Calving-to-first-service interval, d	C-ICF	Interval from calving date to date of first postpartum service in multiparous cows
Multiparous cow gestation length, d	C-GL	Interval from the conception date to the subsequent calving date in multiparous cows
Multiparous cow age at calving, months	C-AC	Interval from birth date to calving date of the corresponding parity
Calving ease, score	CE	Four-category ordinal score based on calving observation and assistance: 1 = unobserved and unassisted; 2 = observed but unassisted; 3 = assisted by farm personnel; and 4 = veterinary assistance or cesarean section [12]
Calf birth weight, kg	CBW	Body weight measured immediately after birth

Note: d, days; kg, kilograms.

**Table 2:** Descriptive statistics of reproductive and calving traits in Jersey cattle.

Trait	No. of Records	Min	Max	Mean	SD
AFS (d)	2716	382	633	480.98	52.24
AFC (month)	2716	21.2	32.25	25.43	2.03
H-GL (d)	2716	242	301	272.93	10.24
H-AIS (times)	2716	1.00	4.00	1.46	0.811
CI (d)	2707	336	476	377.41	29.18
DO (d)	2707	64	198	97.88	29.06
C-AIS (times)	2707	1	6	1.53	0.81
C-ICF (d)	2707	64	130	84	16.11
C-GL (d)	2707	264	294	279.53	4.34
C-AC (month)	2707	28.93	75.77	47.29	9.60
CE (score)	5423	1.00	4.00	2.08	0.342
CBW (kg)	5423	17	41	28.88	4.22

Note: Age at first service, AFS; Age at first calving, AFC; Heifer gestation length, H-GL; Heifer number of services, H-AIS; Calving interval, CI; Days open, DO; Multiparous cow number of services, C-AIS; Calving-to-first-service interval, C-ICF; Multiparous cow gestation length, C-GL; Multiparous cow age at calving, C-AC; Calving ease, CE; Calf birth weight, CBW; No. of records, number of records; SD, standard deviation; Min, Minimum; Max, Maximum.

**Table 3:** Heritability estimates and genetic and phenotypic correlations among reproductive traits in heifers.

Trait	AFS	AFC	H-GL	H-AIS
AFS	0.416 (0.045)	0.602 (0.065) *	−0.119 (0.024) *	0.780 (0.133) *
AFC	0.780(0.164) *	0.380 (0.052)	0.525 (0.068) *	0.862 (0.207) *
H-GL	−0.283(0.031) *	0.055(0.014) *	0.133 (0.040)	0.364 (0.101) *
H-AIS	0.011(0.002)	0.524(0.135) *	0.320(0.073) *	0.135 (0.040)

Note: Heritability estimates are shown on the diagonal, genetic correlations above the diagonal, and phenotypic correlations below the diagonal. All values are presented as estimate (SE), where SE denotes the standard error, * indicates a genetic or phenotypic correlation significantly different from zero at *p* < 0.05. The same convention applies to the following tables. Age at first service, AFS; Age at first calving, AFC; Heifer gestation length, H-GL; Heifer number of services, H-AIS.

**Table 4:** Heritability estimates and genetic and phenotypic correlations among reproductive traits in multiparous cows.

Trait	CI	DO	C-AIS	C-ICF	C-GL	C-AC
CI	0.121 (0.035)	0.781 (0.164) *	0.737 (0.193) *	0.268 (0.051) *	0.075 (0.008) *	0.266 (0.045) *
DO	0.989(0.241) *	0.096 (0.033)	0.756 (0.129) *	0.262 (0.034) *	−0.077 (0.011) *	0.282 (0.071) *
C-AIS	0.773(0.151) *	0.775(0.218) *	0.072 (0.027)	−0.173 (0.018) *	−0.104 (0.016) *	0.245 (0.032) *
C-ICF	0.476(0.062) *	0.492(0.102) *	−0.051(0.014) *	0.150 (0.033)	−0.011 (0.001) *	0.173 (0.029) *
C-GL	0.101(0.013) *	−0.048(0.015) *	0.009(0.001)	−0.095(0.002) *	0.154 (0.032)	0.161 (0.017) *
C-AC	−0.125(0.027) *	−0.140(0.032) *	0.034(0.009)	−0.288(0.053) *	0.092(0.017) *	0.134 (0.026)

Note: Heritability estimates are shown on the diagonal, genetic correlations above the diagonal, and phenotypic correlations below the diagonal. All values are presented as estimate (SE), where SE denotes the standard error, * indicates a genetic or phenotypic correlation significantly different from zero at *p* < 0.05. The same convention applies to the following tables. Calving interval, CI; Days open, DO; Multiparous cow number of services, C-AIS; Calving-to-first-service interval, C-ICF; Multiparous cow gestation length, C-GL; Multiparous cow age at calving, C-AC.

**Table 5:** Heritability estimates and genetic and phenotypic correlations among calving traits in Jersey cattle.

Trait	CE	CBW
CE	0.028 (0.015)	−0.127 (0.017) *
CBW	0.123(0.019) *	0.190 (0.043)

Note: Heritability estimates are shown on the diagonal, genetic correlations above the diagonal, and phenotypic correlations below the diagonal. All values are presented as estimates (SE), where SE denotes the standard error. * indicates a genetic or phenotypic correlation significantly different from zero at *p* < 0.05. The same convention applies to the following tables. Calving ease, CE; Calf birth weight, CBW.

**Table 6:** Genome-wide significant loci and candidate genes located within ±200 kb of significant variants.

Trait	SNP	CHR	Position(bp)	Candidate Gene	Distance (kb)	*p*-Value
AFC	rs385802298	5	91,433,413	*CAPZA3*	97.654	1.48 × 10^−8^
				*PLCZ1*	48.889	
				*PIK3C2G*	within	
H-AIS	rs379656832	4	101,232,152	*DGKI*	within	7.41 × 10^−9^
	rs210480017	25	14,372,905	*MYH11*	120.492	5.37 × 10^−9^
				*CEP20*	95.447	
				*ABCC1*	within	
				*ABCC6*	−100.752	
CI	rs383067957	11	39,069,189	*CCDC85A*	within	2.95 × 10^−8^
CE	rs378446798	6	103,385,828	*CRMP1*	within	2.57 × 10^−8^
				*EVC*	−8.450	
				*EVC2*	−114.783	

Note: CHR, chromosome; Position represents the genomic position of the genome-wide significant variant based on the ARS-UCD1.2 bovine reference genome. Candidate genes were annotated within ±200 kb of each significant variant. Distance represents the distance between the significant variant and the candidate gene. “Within” indicates the variant is located within the gene. Negative and positive values indicate genes located upstream and downstream of the variant, respectively.

## Data Availability

The datasets are available from the corresponding author upon reasonable request.

## Supplementary Materials

The following supporting information can be downloaded at https://www.mdpi.com/article/10.3390/ani16193059/s1, Figure S1: Quantile–quantile (Q–Q) plots of genome-wide association analyses for reproductive and calving traits in Jersey cattle.
