# Supplementary material for: Genetic Parameters and Genome-Wide Association Analysis of Reproductive and Calving Traits in Jersey Cattle
Source: Animals (Basel). 2026 Sep 29;16(19):3059. doi: 10.3390/ani16193059 (PMC13663428; doi:10.3390/ani16193059)
Supplement: Supplementary file 1 [file animals-16-03059-s001.zip › animals-4562003-supplementary.pdf]

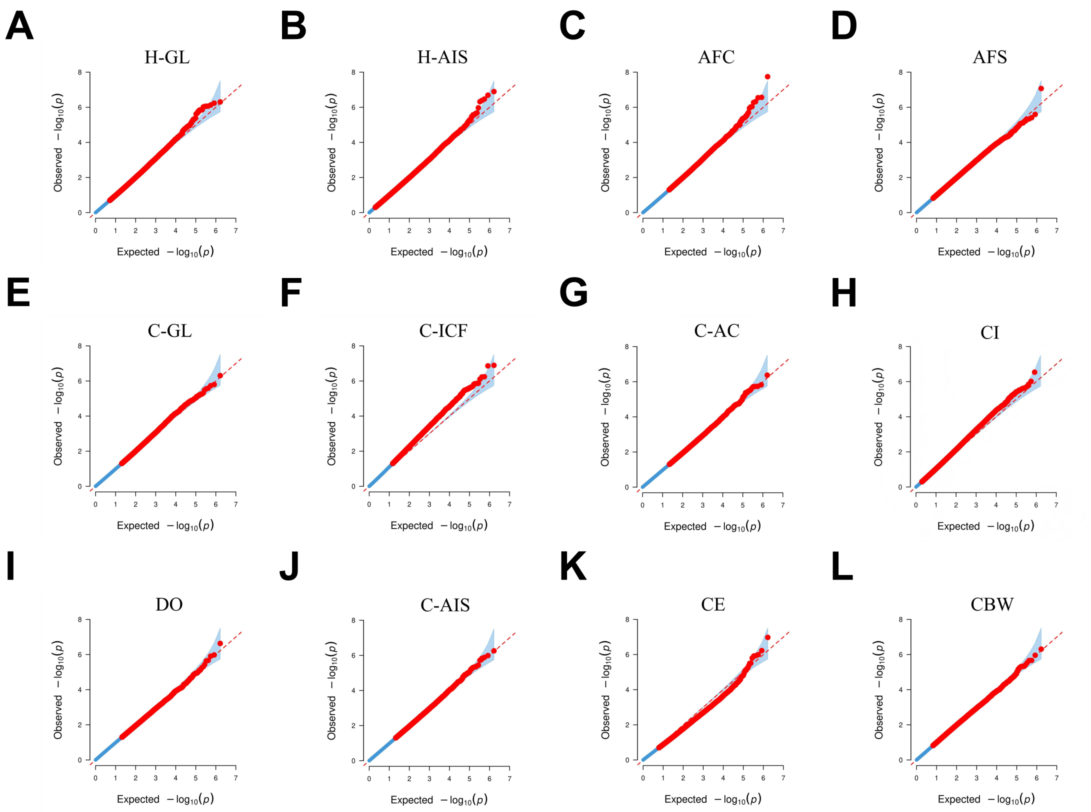

Figure S1. Quantile–quantile (Q–Q) plots of genome-wide association analyses for reproductive and calving traits in Jersey cattle. The plots correspond to H-GL, H-AIS, AFC, AFS, C-GL, C-ICF, C-AC, CI, DO, C-AIS, CE, and CBW, arranged from left to right and top to bottom. The x-axis represents the expected $-\log_{10}(P)$ values under the null hypothesis, and the y-axis represents the observed $-\log_{10}(P)$ values from the GWAS. The diagonal dashed line represents the expected distribution under the null hypothesis, and the shaded region represents the 95% confidence interval.
